# Reconstructive Challenges in Recurrent Nasal Basal Cell Carcinoma: Multidisciplinary Management and Paramedian Forehead Flap Reconstruction in an Elderly Patient

**DOI:** 10.7759/cureus.112932

**Published:** 2026-07-18

**Authors:** Verdy Cendana, Jenisa Amanda Sandiarini Kamayana, Putu Trisna Utami

**Affiliations:** 1 Department of Plastic and Reconstructive Surgery, Bali Mandara Regional General Hospital, Bali, IDN; 2 Department of Plastic Reconstructive and Aesthetic Surgery, Bali Mandara Regional General Hospital, Bali, IDN

**Keywords:** basal cell carcinoma, nasal defect, nasal reconstruction, paramedian forehead flap, recurrent skin cancer

## Abstract

Basal cell carcinoma involving the nose may require extensive surgical excision, resulting in complex defects that present both functional and aesthetic reconstructive challenges, particularly in patients with recurrent disease or previous radiotherapy. We report the case of a 73-year-old woman with a history of recurrent basal cell carcinoma, including prior facial radiotherapy, who presented with a biopsy-proven lesion on the nasal dorsum. Following multidisciplinary evaluation, she underwent wide local excision with histopathological assessment, resulting in a 2 × 2 cm nasal dorsum defect with exposed periosteum and perichondrium. Immediate reconstruction was performed using a paramedian forehead flap to provide adequate soft-tissue coverage. The postoperative course was uneventful, with preserved flap viability and no evidence of ischemia, necrosis, or wound complications. Postoperative wound care included the application of alginate and silicone foam dressings to manage serosanguinous drainage and protect the reconstructed site. Histopathological examination confirmed complete tumor excision with clear margins. At follow-up, the patient demonstrated satisfactory wound healing with favorable functional and aesthetic outcomes and no evidence of early local recurrence. This case highlights the role of multidisciplinary management and paramedian forehead flap reconstruction in achieving successful oncologic and reconstructive outcomes for complex nasal defects following excision of recurrent basal cell carcinoma.

## Introduction

Basal cell carcinoma (BCC) is the most common type of non-melanoma skin cancer, frequently arising in sun-exposed areas of the face, with the nasal region being one of the most commonly involved sites due to chronic ultraviolet radiation exposure. Although BCC typically grows slowly and rarely metastasizes, it can cause significant local tissue destruction if left untreated, especially in high-risk subtypes or recurrent tumors. Management requires complete oncologic excision to achieve tumor-free margins, as confirmed by histological analysis, to minimize the risk of local recurrence [1,2]. Mohs micrographic surgery (MMS) is widely recognized as an important treatment option for high-risk and recurrent facial BCC because it allows precise microscopic margin control while maximizing preservation of uninvolved tissue. Nevertheless, treatment selection should also consider lesion characteristics, recurrence status, multidisciplinary evaluation, and the anticipated reconstructive requirements following tumor excision [3].

After wide oncologic resection, reconstruction of nasal defects poses a significant surgical challenge due to the nose’s central functional and aesthetic roles. Small and superficial defects may be amenable to secondary healing or skin grafts, but larger or deeper defects involving multiple nasal subunits, such as the dorsum, tip, and structural framework, often necessitate local or locoregional flap reconstruction. The paramedian forehead flap (PFF), based on the supratrochlear artery, remains the “workhorse” for large or complex nasal reconstructions because of its robust vascular supply, excellent tissue match, and versatility in restoring both form and contour [1,2]. The choice of reconstructive technique should be individualized based on the size, depth, anatomical location, and tissue components of the defect, as well as patient-related factors. Furthermore, the Burget and Menick nasal aesthetic subunit principle serves as a fundamental guide for reconstruction, recommending consideration of subunit replacement when a substantial portion of a nasal aesthetic subunit is involved to optimize contour, symmetry, and scar camouflage while preserving nasal function [4].

In elderly patients with comorbidities such as hypertension and a history of multiple BCCs, careful multidisciplinary planning is essential to optimize oncologic and reconstructive outcomes. The PFF is performed in staged procedures to restore the nasal skin envelope and, when necessary, combined with cartilage grafts to reconstitute structural support, thereby achieving both functional and cosmetic goals [5].

## Case presentation

A 73-year-old woman was admitted for preoperative evaluation before a planned combined oncologic and plastic reconstructive procedure. She was diagnosed with biopsy-proven BCC of the nasal dorsum and scheduled for wide local excision with immediate reconstruction. The patient reported a slowly progressive nasal lesion over approximately one year, initially brownish in appearance and becoming erythematous over the preceding four months. She denied bleeding, pruritus, pain, or unintentional weight loss. Histopathological examination from a biopsy performed two months prior confirmed BCC (Figure 1).

**Figure 1:**
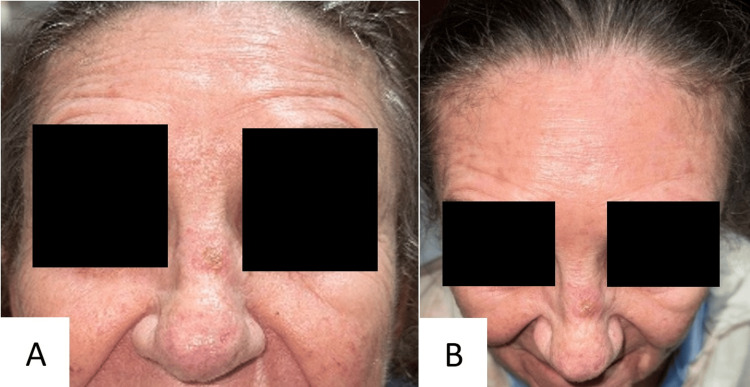
Clinical presentation. (A) Frontal view and (B) bird’s-eye view showing a raised, erythematous lesion with an irregular border and central ulceration, measuring approximately 0.6 × 0.6 cm on the nasal dorsum overlying the nasal bone, surrounded by a pinkish margin.

Her medical history was notable for hypertension and multiple episodes of BCC of the face over the past two decades. The most recent lesion involved the right cheek in 2022 and was treated with 24 sessions of radiotherapy. Current medications included lisinopril 10 mg daily, sertraline 10 mg daily, zolpidem, and atorvastatin. No drug allergies were reported. On physical examination, the patient was in good general condition with stable vital signs. Local examination revealed a well-demarcated, erythematous ulcerative lesion measuring approximately 0.6 × 0.6 cm on the nasal dorsum overlying the nasal bone, surrounded by a pinkish margin. Laboratory investigations demonstrated normal hematologic and biochemical parameters. Chest radiography showed atherosclerotic changes of the aorta without evidence of pulmonary or skeletal metastasis (Figure 2).

**Figure 2:**
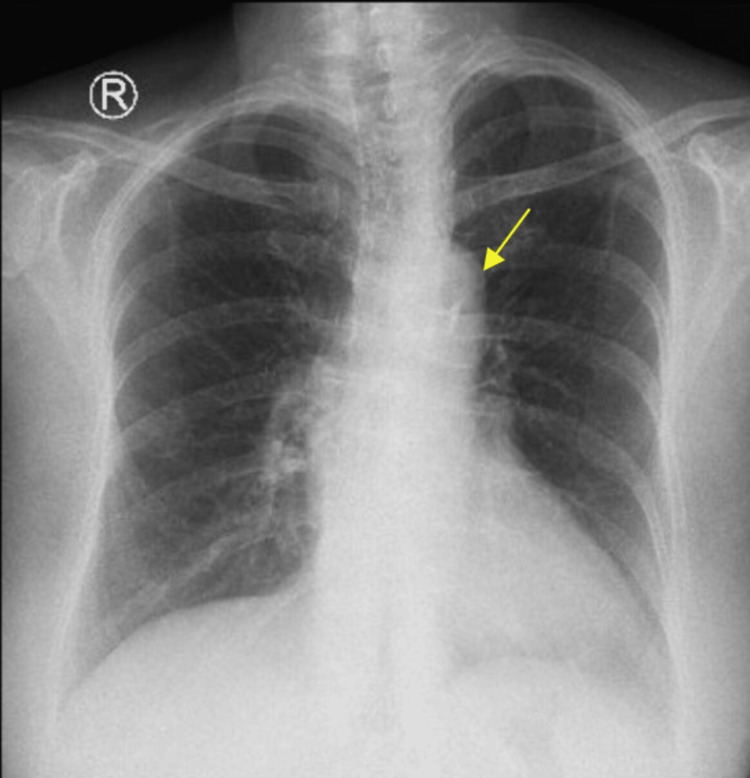
Preoperative chest radiograph. Posteroanterior chest radiograph demonstrating no radiographic evidence of pulmonary or osseous metastasis. Incidental atherosclerotic calcification of the aortic knob is noted (yellow arrow).

Multidisciplinary evaluation confirmed the diagnosis and surgical plan. The oncology team recommended wide local excision, while anesthesiology classified the patient as American Society of Anesthesiologists (ASA) physical status II [6]. Internal medicine assessment concluded that the patient was medically optimized for surgery. The reconstructive plan included a PFF for nasal defect coverage, with contingency options for additional local flaps or structural grafting depending on the intraoperative extent of the defect (Figure 3).

**Figure 3:**
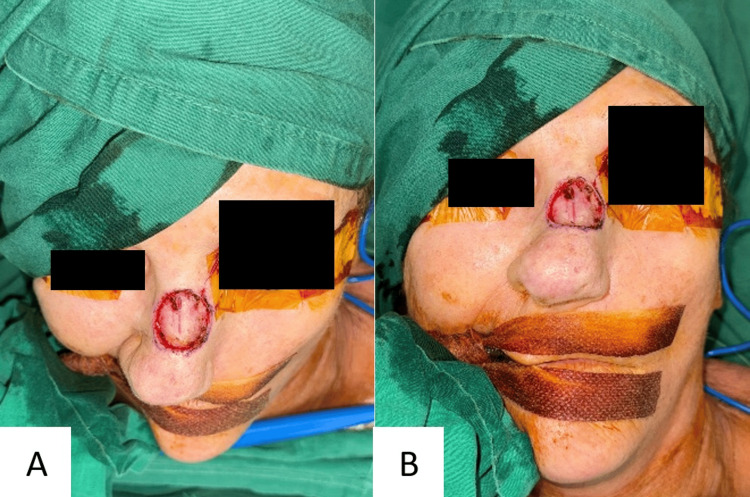
Preoperative assessment and surgical defect. (A) Bird’s-eye view and (B) frontal view demonstrating the nasal dorsum following wide local excision performed by the oncologic surgeon, resulting in a 2 × 2 cm defect with exposed periosteum and perichondrium.

The patient was placed in a supine position under general anesthesia. After skin preparation with povidone-iodine and sterile draping, a wide local excision of the nasal lesion was performed by the surgical oncology team, and the specimen was sent for histopathological examination. Final histopathological examination of the excised specimen confirmed nodular BCC. Microscopically, the dermis contained a neoplastic basaloid cell proliferation forming solid nodular structures infiltrating the surrounding connective tissue. Peripheral palisading and cleft artifact were identified, while the central portion showed haphazardly arranged tumor cells with round-to-oval hyperchromatic nuclei and scant cytoplasm. The closest surgical margin was 1 mm at the superior margin and 1 mm at the deep margin, whereas the inferior margin was 4 mm, the right lateral margin was 4 mm, and the left lateral margin was 3 mm. The final pathological stage was reported as pT1 pNx pMx. The operative field was irrigated with normal saline, and the procedure was continued by the plastic surgery team after informed consent had been confirmed. Intraoperatively, the excision resulted in a defect sized 2 cm × 2 on the nasal dorsum with exposure of the periosteum and perichondrium. Following resection, a 2 × 2 cm nasal dorsal defect with exposed periosteum and perichondrium was identified. Although the underlying bone and cartilage were not exposed, the defect involved a cosmetically and functionally important nasal subunit and required restoration of nasal contour and skin envelope quality. The PFF was selected because it provides reliable vascularity, adequate tissue volume, and superior color and texture match for nasal reconstruction, thereby supporting both functional restoration and aesthetic outcome. Reconstruction was performed using a right PFF designed along the course of the supratrochlear artery. The flap was elevated according to the standard PFF technique described by Menick. Elevation was performed in the subcutaneous plane over the distal portion of the flap and transitioned to a subgaleal plane proximally, while the vascular pedicle was carefully preserved. Thus, the flap was not elevated entirely in the subperiosteal plane. The distal portion of the flap was then transposed and inset to cover the nasal dorsum defect without tension. Meticulous hemostasis was achieved before layered closure. No drain was placed, and the residual donor-site defect was dressed with alginate. The raw surface on the posterior side of the forehead flap was covered with an alginate dressing. The operation was then concluded without intraoperative complications (Figure 4).

**Figure 4:**
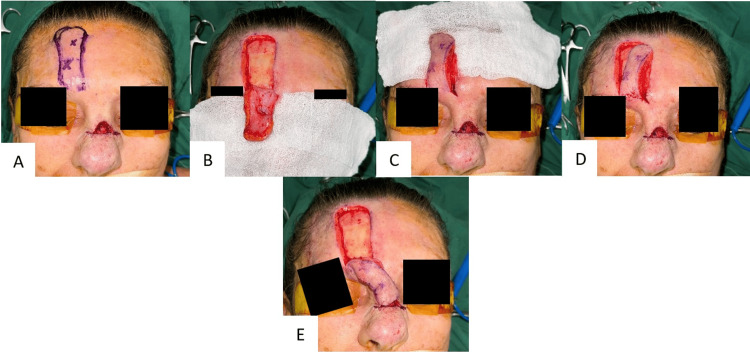
Intraoperative reconstruction using a right paramedian forehead flap. (A) Flap design along the course of the right supratrochlear artery. (B) Flap elevation in the subperiosteal plane with preservation of the vascular pedicle. (C, D) Elevated flap and nasal defect following wide local excision. (E) Transposition and inset of the distal flap to reconstruct the nasal dorsum defect.

In the immediate postoperative period, the patient was alert and hemodynamically stable. Vital parameters were within acceptable limits, with a slightly elevated blood pressure of 158/87 mmHg. Close postoperative monitoring was performed with particular attention to bleeding from the forehead flap pedicle, hematoma formation, flap color, capillary refill, and early signs of vascular compromise. This is especially important because the highly vascularized pedicle of the PFF predisposes patients to early postoperative bleeding, making prompt recognition and management essential to preserve flap viability [7]. On postoperative day one, the patient experienced mild bleeding in the form of oozing from the forehead flap, which was managed conservatively with local wound care. Local examination of the nasal-forehead region demonstrated minimal serosanguinous oozing from the forehead flap without evidence of active bleeding, venous congestion, or flap ischemia. Postoperative management consisted of intravenous ceftriaxone (2 g once daily), intravenous dexamethasone (5 mg twice daily), oral sucralfate syrup administered three times daily for dyspepsia, and analgesics according to the anesthesiologist. The patient was permitted a regular diet, with preserved bowel sounds, and was instructed to maintain a supine position with the head elevated to reduce swelling and risk of bleeding. Local flap warming was applied using a therapeutic lamp positioned approximately 50 cm from the forehead. The surgical dressing was kept clean and dry, with wound care performed as indicated. During the first postoperative day, local hemostasis and exudate control were achieved using an alginate dressing to absorb oozing blood and wound exudate, followed by coverage with a silicone foam dressing to provide atraumatic protection of the flap and additional exudate management (Figure 5).

**Figure 5:**
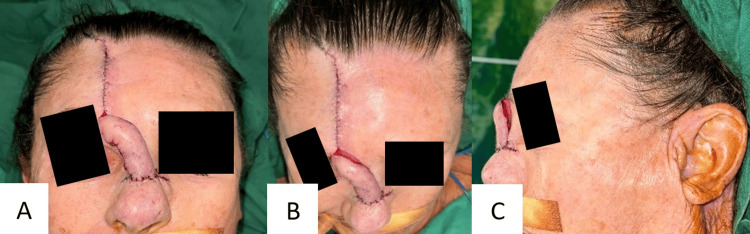
Immediate postoperative assessment. (A) Frontal view, (B) bird’s-eye view, and (C) lateral view demonstrating complete coverage of the nasal defect with the paramedian forehead flap. The flap was well perfused and viable, with satisfactory hemostasis and no evidence of immediate postoperative complications.

The patient was followed postoperatively on an outpatient basis. The patient remained clinically stable with no significant complaints, and hypertension was controlled with medication. Local examination revealed a viable nasal flap with intact sutures, no dehiscence, and no evidence of venous congestion, ischemia, or infection; the frontal donor site healed well, with only minimal exudate noted. Postoperative care consisted of continued local wound management and staged suture removal, with planned assessment of flap viability before pedicle division, which would be performed once adequate vascular maturation was confirmed (Figure 6).

**Figure 6:**
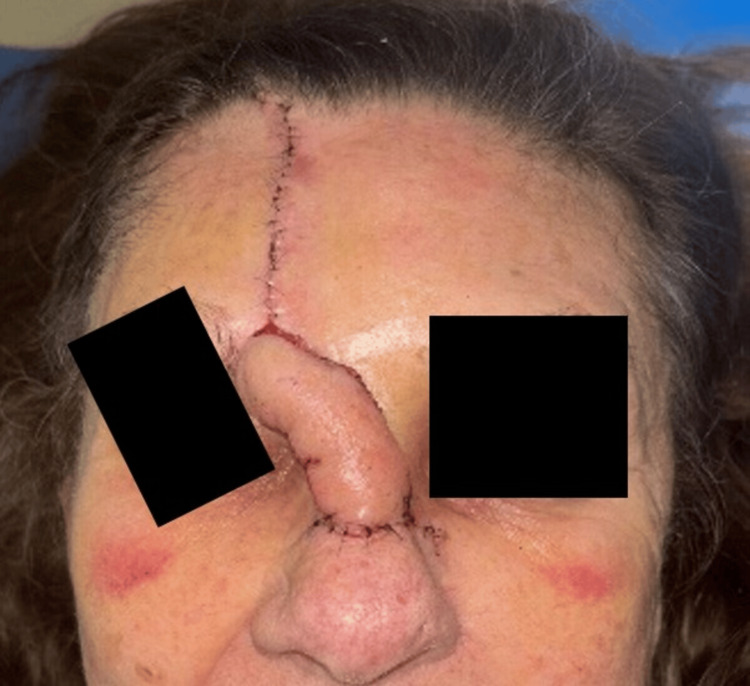
Postoperative day five following reconstruction with a paramedian forehead flap. The nasal forehead flap remained viable with intact sutures and no evidence of necrosis, wound dehiscence, venous congestion, ischemia, hematoma, or surgical site infection. The frontal donor site demonstrated satisfactory healing with minimal serous exudate.

The patient was followed up regularly in the outpatient clinic after the initial wide excision and reconstruction with a PFF performed in January 2026. Close postoperative monitoring was performed with particular attention to bleeding from the forehead flap pedicle, hematoma formation, flap color, capillary refill, and early signs of vascular compromise. On postoperative day 25 (February 2026), approximately 3.5 weeks after the initial reconstruction, a temporary pedicle occlusion (flap viability) test was performed before the planned pedicle division to assess vascular autonomy of the flap. A modified latex glove strip was gently applied around the pedicle to temporarily occlude vascular inflow for approximately 15 minutes while the flap was observed for changes in color and other clinical indicators of perfusion. Mild bluish discoloration developed at the distal portion of the flap, indicating incomplete vascular autonomy from the recipient bed. Consequently, pedicle division was postponed and reassessed one week later, when adequate flap perfusion was confirmed, allowing safe progression to the second-stage procedure (Figure 7). The patient then underwent stage-2 reconstruction with flap division approximately four weeks after the first operation (Figure 8). The immediate postoperative period was clinically stable without complications, with minimal pain controlled by analgesics and stable vital signs. The flap remained viable, with good color, warmth, capillary refill, intact sutures, and no evidence of dehiscence, infection, or significant venous congestion. The frontal donor site healed well under local wound care, with only minimal exudate noted during follow-up. Sutures were removed gradually as healing progressed, and the patient was instructed to continue wound care, maintain a high-protein diet, keep the head elevated, and avoid pressure or contamination of the flap. At the latest follow-up, both the nasal reconstruction site and the frontal donor site showed good healing, and the patient remained clinically stable with controlled hypertension (Figure 9).

**Figure 7:**
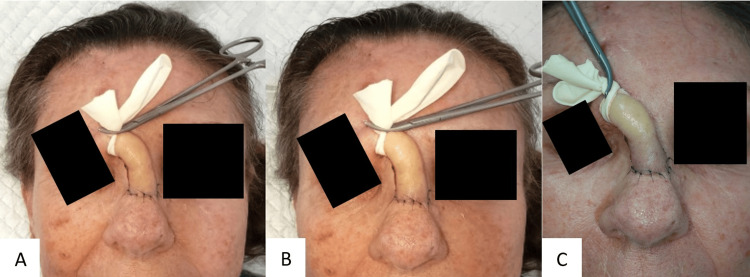
Flap viability test. (A) At postoperative week one, mild bluish discoloration was observed at the distal portion of the flap, suggesting suboptimal perfusion. (B) At postoperative week two, signs of suboptimal perfusion persisted. (C) Therefore, the procedure was postponed and reassessed one week later; the discoloration had improved, supporting the decision to proceed with second-stage flap division in the fourth postoperative week.

**Figure 8:**
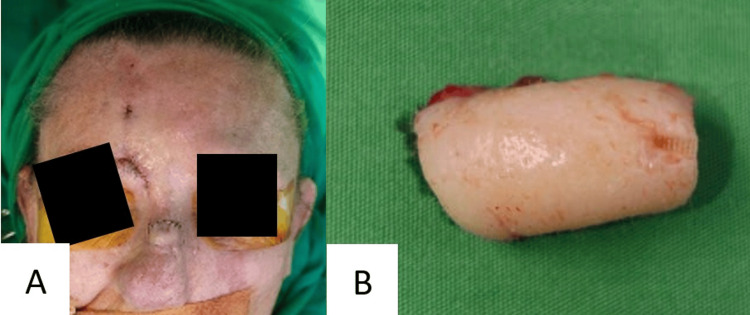
Intraoperative view of the second-stage procedure demonstrating division and inset of the paramedian forehead flap. (A) Frontal view following division and inset of the flap with the remaining sutures in place. (B) Pedicle division after flap separation from the forehead donor site.

**Figure 9:**
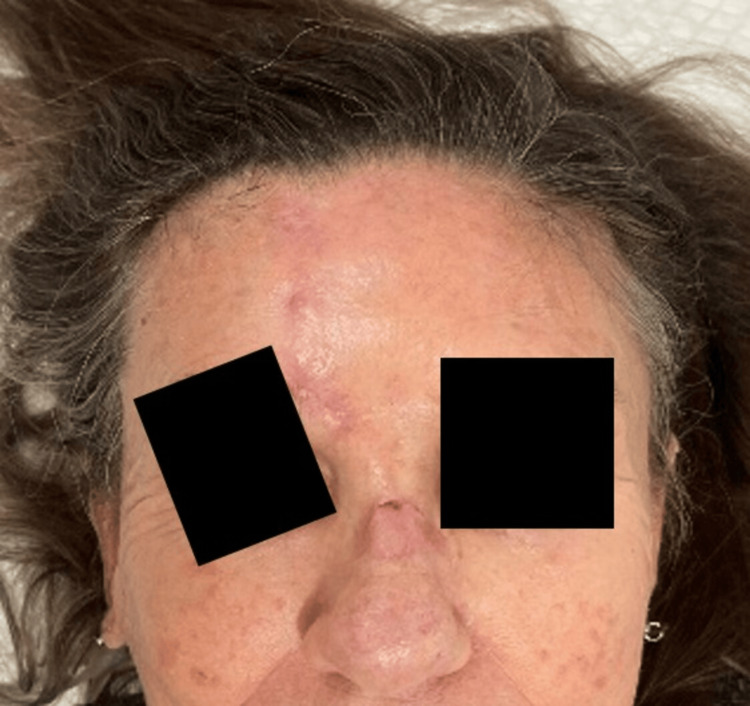
Postoperative follow-up after the second-stage procedure showing a well-vascularized and viable flap with satisfactory contour and aesthetic outcome, with the patient reporting high satisfaction.

## Discussion

Recurrent BCC of the nose represents a distinct oncologic challenge because lesions in high-risk facial zones are more likely to exhibit subclinical extension and to demonstrate a higher recurrence rate after inadequate excision. For recurrent lesions, especially in central facial subunits, definitive oncologic management prioritizes complete excision with histologic margin control. This is due to the primary BCCs having higher cure rates and lower metastatic rates than other types of skin tumors, including recurrent skin tumors. In this patient, the history of multiple prior BCCs and a biopsy-proven nasal lesion justifies a surgical strategy aimed at wide excision with margin assessment before definitive reconstruction [8,9]. MMS is recognized as an important contemporary treatment option for high-risk and recurrent facial BCC because it provides precise microscopic margin control while maximizing preservation of uninvolved tissue. Nevertheless, treatment selection should be individualized according to lesion characteristics, recurrence status, multidisciplinary evaluation, and the anticipated reconstructive requirements following tumor excision. In the present case, after multidisciplinary discussion involving the surgical oncology and plastic surgery teams, wide local excision with histopathological margin assessment was considered an appropriate oncologic approach because it allowed complete tumor removal with subsequent confirmation of margin status and facilitated coordinated definitive reconstruction [10].

The choice of reconstructive technique for nasal defects should be individualized according to defect size, depth, anatomical location, involvement of structural support, and patient-related factors. Small superficial defects may be managed with secondary intention healing, primary closure, skin grafts, or local flaps such as bilobed, nasolabial, or dorsal nasal flaps. However, larger, deeper, or multi-subunit defects generally require interpolated flaps such as the PFF. The PFF remains the reconstructive “workhorse” for medium-to-large nasal defects because it provides well-vascularized, sensate tissue with excellent color and texture match and sufficient bulk for three-dimensional contour restoration. In the present case, wide local excision resulted in a 2 × 2 cm defect on the nasal dorsum with exposure of the periosteum and perichondrium. Although the underlying bone and cartilage were not exposed, the defect involved a cosmetically and functionally important nasal subunit and required restoration of nasal contour and skin envelope quality. Therefore, simpler reconstructive options were considered less optimal because they might not provide sufficient tissue volume, contour restoration, or aesthetic match for this visible facial region [11].

The PFF was selected because it provided reliable vascularity, adequate tissue volume, and superior color and texture match for reconstruction of the nasal dorsum, thereby supporting both functional restoration and aesthetic outcome. The robust axial supply from the supratrochlear artery permits reliable vascularization to support flap viability. PFF procedure is commonly done in two to three stages; the first stage involves transfer of the forehead flap with an intact supratrochlear pedicle to resurface the nasal defect; the second stage is typically performed two to three weeks later, for pedicle division and inset. A third-stage procedure may be required for debulking/thinning, and placement of cartilage support occurs between transfer and final division to improve contour if needed. Traditionally, the time between the first and second stage is around two to three weeks before pedicle takedown to allow revascularization from the recipient bed, and proponents of the three-stage approach argue the extra intermediate stage yields thinner, more refined results and can reduce the need for later revisions [11]. However, several groups have reported safe accelerated/early division protocols, retrospective series and case reports describe complete pedicle division as early as one week in selected patients, and prospective work has used adjuncts such as indocyanine green (ICG) angiography to document flap perfusion before early takedown. These studies suggest early division can improve patient quality of life (less time with a bulky pedicle) without increasing necrosis when patients are carefully selected and perfusion is confirmed [12-14]. Early (approximately one week) pedicle division may be feasible in carefully selected patients with small or partial-thickness defects and a well-vascularized recipient site, and when objective perfusion assessment (ICG) is available; but for large, complex full-thickness reconstructions or when maximal aesthetic refinement is the priority, the standard two to three-week or three-stage approach remains the more conservative choice. In the present case, however, flap division was postponed until the fourth postoperative week because flap viability testing at postoperative day 14 still demonstrated mild bluish discoloration at the distal flap, indicating that vascular maturation had not yet fully stabilized. After one additional week, the bluish discoloration had begun to fade, supporting safer pedicle division and second-stage reconstruction. Thus, although early takedown may be feasible in selected patients, a delayed four-week interval was chosen in this case to ensure adequate flap perfusion and minimize the risk of distal ischemia or necrosis [15].

The usage of PFF also allows a myriad of technique variations, such as the incorporation of cartilage or bone grafts for structural support. Given the lesion’s location on the dorsum and the potential for full-thickness resection, selection of a PFF is consistent with contemporary reconstructive algorithms that favor local axial flaps over grafting for complex nasal subunit reconstruction. General anesthesia was selected because the procedure involved a combined oncologic and reconstructive operation, including wide local excision, PFF design and elevation, flap transposition, inset, and donor-site management. The anticipated operative duration and the need for meticulous intraoperative control favored the use of general anesthesia. Furthermore, general anesthesia provided optimal patient comfort, immobility, and airway control, which were considered important for the precision required during flap elevation and inset [16,17].

Patient-specific factors, such as advanced age, hypertension, and prior radiotherapy to the face, must inform perioperative planning because they can largely influence wound healing, flap survival, and complication rates. Although forehead flaps have high overall success rates, prior radiation can increase fibrosis, impair microvascularity of recipient beds, and raise the likelihood of delayed healing or flap atrophy; therefore, preoperative optimization (blood-pressure control, correction of anemia if present, glycemic control where relevant) and meticulous intraoperative technique to preserve pedicle perfusion are essential. Multidisciplinary discussion with internal medicine and anesthesia to minimize cardiometabolic risks in the perioperative period helps reduce complication risk in elderly patients undergoing staged reconstruction [18,19].

The multidisciplinary team also considered the potential role of targeted therapy with vismodegib (Erivedge), a hedgehog pathway inhibitor that has emerged as an important treatment option for patients with locally advanced or metastatic BCC that is unsuitable for curative surgery or radiotherapy. However, in this patient, vismodegib was not indicated because complete oncologic excision with histologically negative margins was achieved, and definitive reconstruction was feasible. Current international guidelines recommend hedgehog inhibitors primarily for BCC that is not amenable to curative surgery and/or radiotherapy, rather than as routine adjuvant therapy following successful complete resection [20].

Postoperative warming of local flaps using therapeutic heat lamps applied at a calibrated distance has been used to promote local vasodilation, restore microcirculatory flow, and support postoperative flap perfusion and viability. Methods reported in the literature include forced-air systems, heated blankets, and therapeutic lamps, and when employed after transfer, lamps can help maintain skin temperature, reduce vasoconstriction from ambient cooling, and potentially decrease marginal ischemia. However, unregulated lamp use carries a documented risk of low-temperature burns to recently reconstructed tissues, so lamp power, lamp-to-skin distance, exposure duration, and continuous observation must follow manufacturer recommendations and institutional protocols. Consequently, best practice for postoperative heat-lamp warming is to use regulated warming devices when available or to adopt a calibrated lamp setup with explicit distance/power guidelines, continuous monitoring of flap temperature and skin integrity, and thorough documentation of the warming technique and safety measures in clinical reports [21].

Finally, staged PFF reconstruction combined with appropriate structural grafting and close oncologic surveillance offers a balance between tumor clearance and functional/aesthetic rehabilitation. An initial stage focused on oncologic resection and reliable soft-tissue coverage should be followed by staged pedicle division and refinement, with cartilage or bone grafting as indicated to restore the nasal framework; patient counseling about the multi-stage nature and expected timeline of reconstruction is critical. Long-term follow-up is required to monitor for local recurrence, evaluate airway function, and document cosmetic outcomes. Published series support favorable success and satisfaction rates for staged PFF reconstructions when performed within a multidisciplinary care pathway [7,22].

## Conclusions

This case illustrates the multidisciplinary decision-making process in the management of recurrent BCC of the nasal dorsum in an elderly patient. Wide local excision with histopathological margin assessment provided oncologic control, while staged reconstruction using a PFF enabled restoration of nasal contour and function in a complex post-excisional defect with exposed periosteum and perichondrium. Although the PFF is a well-established reconstructive technique, this report highlights its practical application in achieving reliable soft-tissue coverage and satisfactory functional and aesthetic outcomes after wide excision. Careful perioperative optimization and close postoperative monitoring were important in supporting flap viability and minimizing complications in this patient with comorbidities and prior treatment history.
